# A novel insertional allele of the *CG18135* gene is associated with severe mutant phenotypes in *Drosophila melanogaster*

**DOI:** 10.3389/fgene.2024.1355368

**Published:** 2024-06-17

**Authors:** Attila Cristian Ratiu, Adrian Ionascu, Alexandru Al. Ecovoiu

**Affiliations:** ^1^ Drosophila Laboratory, Faculty of Biology, University of Bucharest, Bucharest, Romania; ^2^ Academy of Romanian Scientists, Ilfov, Bucharest, Romania

**Keywords:** CG18135, P{lacW} insertion, *Drosophila melanogaster*, mutant phenotype, lethality, gene expression

## Abstract

*Drosophila melanogaster* has been at the forefront of genetic studies and biochemical modeling for over a century. Yet, the functions of many genes are still unknown, mainly because no phenotypic data are available. Herein, we present the first evidence data regarding the particular molecular and other quantifiable phenotypes, such as viability and anatomical anomalies, induced by a novel *P{lacW}* insertional mutant allele of the *CG18135* gene. So far, the *CG18135* functions have only been theorized based on electronic annotation and presumptive associations inferred upon high-throughput proteomics or RNA sequencing experiments. The descendants of individuals harboring the *CG18135* ^*P{lacW}CG18135*^ allele were scored in order to assess mutant embryonic, larval, and pupal viability *versus* Canton Special (CantonS). Our results revealed that the homozygous *CG18135* ^*P{lacW}CG18135*^/*CG18135* ^*P{lacW}CG18135*^ genotype determines significant lethality both at the inception of the larval stage and during pupal development. The very few imago escapers that either breach or fully exit the puparium exhibit specific eye depigmentation, wing abnormal unfolding, strong locomotor impairment with apparent spasmodic leg movements, and their maximum lifespan is shorter than 2 days. Using the quantitative real-time PCR (qRT-PCR) method, we found that *CG18135* is upregulated in male flies, but an unexpected gene upregulation was also detected in heterozygous mutants compared to wild-type flies, probably because of regulatory perturbations induced by the *P{lacW}* transposon. Our work provides the first phenotypic evidence for the essential role of *CG18135*, a scenario in accordance with the putative role of this gene in carbohydrate-binding processes.

## 1 Introduction

*Drosophila melanogaster* has been studied for over a century, serving as a prominent model organism in genetics, biochemistry, molecular biology, and bioinformatics. Even though the *D. melanogaster* genome was sequenced in 2000 (Adams et al., 2000), there are no available phenotypic data to support the characterization and annotation of a number of computed genes (CGs). One example is *CG18135*, which was identified in 2004 via computational techniques (Gramates et al., 2022).

*CG18135* is located on 3L:18990026–18998283 on the genomic minus strand and is known to encode five transcripts that are translated into proteins having between 636 and 770 amino acids. *CG18135* sequence is not only conserved in higher eukaryotes such as fish, reptiles, birds, and mammals but also in invertebrates and yeasts, according to DIOPT v9.1 (Hu et al., 2011).The *CG18135* gene is orthologous to human *glycerophosphocholine phosphodiesterase 1* (*GPCPD1*). *CG18135* gene function was not previously genetically studied in *D. melanogaster*, but the associated protein was evidenced to facilitate myosin filament binding during embryogenesis via the interaction with the Myo10A protein at the plasma membrane level (Liu et al., 2008) and participate in glycerophospholipid catabolism (Gaudet et al., 2011).

*CG18135* has been documented to participate in embryogenesis and to be active through all the developmental stages in wild-type individuals of *D. melanogaster*. Increased levels of CG18135 RNA were identified in both early (0–4 h) and late (20–24 h) embryonic stages (Fisher et al., 2012). Highly increased levels of CG18135 RNA were found in pupae and adult flies compared to embryos, suggesting that the gene plays an important role beyond the embryonic stage (Casas-Vila et al., 2017). In previous studies, it has been shown that 1-week-old adult male flies have significantly higher *CG18135* gene expression than similarly aged female flies (Brown et al., 2014; Casas-Vila et al., 2017).

Several transposon insertions in *CG18135* were reported in FlyBase (FB 2023_05), namely, nine *P* elements, one *PiggyBac* (*PBac*), and two *Minos*-mediated integration cassette (MiMIC)-derived artificial mobile elements (Buels et al., 2016; Gramates et al., 2022; FlyBase Curators, 2004). The *P* and *PBac* artificial transposons as well as one *MiMIC* (Venken et al., 2011) are located in the 5’ half of *CG18135*, defining a local insertional hotspot with a total of 11 transposons in a 1267-bp span. Interestingly, none of them determine noticeable phenotypic consequences.

During one of our previous experiments, we achieved mutagenesis mediated by the mobilization of the *P{lacW}γCop* ^*057302*^ transposon (Ratiu et al., 2008) located in the 5′UTR of *γCop*. The idea backing this study was that transposon mobilization starting from an essential gene, such as *γCop*, would lead to conservative reinsertions in other essential genes, eventually functionally linked. One of the lines generated in this experiment is MZ4CM3, which contains both *P{lacW}γCop* ^*057302*^ and *P{lacW}* ^*CG18135.MZ4CM3*^ transposons located on the 3R and 3L chromosomes, respectively. The latter *P{lacW}* resides in the first intron of the CG18135-RB transcript at the genomic coordinates 3L:18996755–18996762 (GenBank/NCBI accession number: HQ695001.1). This nucleotide interval is the one with the maximum resolution for *P{lacW}* ^*CG18135.MZ4CM3*^ mapping, as debated by Ecovoiu et al. (2016), due to the elusive nature of the octet duplication. Since *P{lacW}γCop* ^*057302*^ determines embryonic lethality in homozygous conditions (Deak et al., 1997; Ecovoiu et al., 2002), the actual phenotypic effect of *P{lacW}* ^*CG18135.MZ4CM3*^ was impossible to assess as long as the two alleles were linked. We successfully performed a series of genetic crosses, allowing us to separate the *P{lacW}γCop* ^*057302*^ and *P{lacW}* ^*CG18135.MZ4CM3*^ transposons by crossing-over and constructing the *CG18135* ^*P{lacW}CG18135*^-Sep1H line, which contains only the novel *P{lacW}* insertion in *CG18135*. To out-cross *P{lacW}* ^*CG18135.MZ4CM3*^, we used the w^1118^ strain; thus, the third chromosome containing solely *P{lacW}* ^*CG18135.MZ4CM3*^ has a mosaic structure consisting of fragments from both the original and isogenic w^1118^ genotypes. Considering the new chromosome makeup, we assumed that the specific phenotypes of the *CG18135* ^*P{lacW}CG18135*^-Sep1H line are caused by perturbations of *CG18135* gene functions. Accordingly, the reversion test confirmed that *P{lacW}* ^*CG18135.MZ4CM3*^ excision rescues the specific mutant phenotypes.

Herein, we report the first phenotypic and functional characterization of this original insertional allele of the *CG18135* gene, symbolized *CG18135* ^*P{lacW}CG18135*^. Our main objectives were to investigate the viability of heterozygous and homozygous *CG18135* ^*P{lacW}CG18135*^ mutant flies during various developmental stages, score the phenotypic features of the elusive homozygous mutant adult escapers, and evaluate the *CG18135* expression level in adult male and female mutant heterozygotes. Moreover, we used a set of bioinformatics tools in order to investigate the putative regulatory consequences induced by *P{lacW}* ^*CG18135.MZ4CM3*^.

## 2 Materials and methods

### 2.1 *D. melanogaster* lines and rearing

In this study, two distinct *D. melanogaster* lines were used: the reference Canton Special (CantonS) line and the new mutant line harboring the *CG18135* ^*P{lacW}CG18135*^ insertional allele (GenBank/NCBI accession number: HQ695001.1) in either homozygous or heterozygous conditions. The chromosome containing the mutant allele was kept over two distinct balancer chromosomes (TM3, *Ser*, *GFP*, *e* or TM6B, *Tb*, *Hu*, *e*), and the respective derived lines were symbolized as follows:

• *y* ^−^, *w* ^−^; *CG18135* ^*P{lacW}CG18135*^ *-Sep1H*/TM3, *Ser*, *GFP*, *e*; in brief, *CG18135-P{lacW}*/GFP;
• *w* ^*-*^; *CG18135* ^*P{lacW}CG18135*^ *-Sep1H*/TM6B, *Tb*, *Hu*, *e*; in brief, *CG18135-P{lacW}*/TM6B.

The GFP construct activation is based on the Gal4–UAS system and produces a green fluorescence that is visible under UV excitation, starting with 10–12 h-old embryos (Rudolph et al., 1999).

The flies were placed in glass vials on standard wheat–banana–yeast–agar-based growth medium and kept under 12h/12 h light/dark cycles with a constant temperature of 18°C–19°C and humidity of 20%–60%. Under experimental conditions, the flies were raised in plastic vials or Petri dishes on the standard growth medium and, alternatively, on the commercially available Grape Agar medium (Genesee Scientific).

### 2.2 Viability experiment

Flies from the *CG18135-P{lacW}*/GFP, *CG18135-P{lacW}*/TM6B, and CantonS lines were placed for a maximum of 24 h in 50-mm embryo collection cages (FlyStuff) coupled with Petri dishes containing Grape Agar medium (roughly 100 male flies and 100 female flies/cage). Throughout the developmental stages, the fluorescent phenotype, determined by the presence or absence of the *GFP* construct, was assessed using an Olympus U-RFL-T UV Lamp. More precisely, the *CG18135* ^*P{lacW}CG18135*^/GFP individuals emit a specific green fluorescence that is evident beginning with the late embryonic stage. GFP/GFP individuals die as embryos, while individuals having the *CG18135* ^*P{lacW}CG18135*^/*CG18135* ^*P{lacW}CG18135*^ genotype can reach late embryonic and subsequent developmental stages. Under the UV lamp, the latter individuals release a pale-blue characteristic auto-fluorescence, clearly different from that of the GFP construct.

We performed two distinct viability assessments. Both experiments started by placing the randomly collected embryos from each considered line, either individually (first experiment) or in groups of five (second experiment), in 24-well plates on a standard medium. During the first evaluation involving all three lines, the focus was on monitoring the overall embryonic development, and for this purpose, each well was regularly inspected until second or third instar larvae were noticed and phenotypically scored. In the second round of experiments, we focused on the surveillance of mutant homozygous individual development and used only the *CG18135-P{lacW}*/GFP line. The surviving non-GFP larvae from each initial group of five embryos were removed and individually placed in 48-well plates containing standard medium and then inspected at least two times a day during their larval and pupal development. The rare adult individuals that emerged from the puparium were phenotypically scored and pictured, and short videos were taken.

### 2.3 Gene expression experiment

The relative gene expression of *CG18135* was measured in 50-h-old adult female flies and male flies from the *CG18135-P{lacW}*/TM6B mutant line and the CantonS control line. Biological triplicates consisting of 20 flies from each sex were treated for RNA stabilization using the DNA/RNA Shield Kit, and then RNA extraction was achieved using the RNeasy Mini Kit (Promega). cDNA conversion was performed by combining the SuperScript II Reverse Transcriptase (Invitrogen) with the Reverse Transcription System Kit (Promega) using random primers and adjusting the RNA volumes based on RNA concentrations.

qRT-PCR amplifications were done in technical triplicates using the SYBR Green ROX qPCR Master Mix Kit (QIAGEN) with newly designed *CG18135* primers (Table 1) and available *RpL32* endogenous primers (Fiumera et al., 2005). Each qRT-PCR reaction tube contained 20 ng of cDNA, 0.5 µM of primers (for each forward/reverse primer), 1X master mix solution, and PCR-grade water up to 20 µL of the final reaction volume. The cycle threshold (Ct) values were detected at a fluorescence threshold Rn = 0.2 (raw Ct data are available in Supplementary Material S1).

**TABLE 1:** Characteristics of the primers used for gene expression analysis with qRT-PCR. The primers’ melting temperatures were provided by Integrated DNA Technologies for *CG18135* and Eurogentec for *RpL32*.

Gene	Orientation	Nucleotide sequence	Molecular mass (g/mol)	Melting temperature (°C)	CG%
*CG18135*	F	5′ ATC​TTT​CAC​ATC​ACG​CTG​CC 3′	5,987	55.8	50
	R	5′ GAC​ATA​GGG​AAG​CCT​CAG​CC 3′	6,136	57.5	60
*RpL32*	F	5′ CGG​CTT​CAA​GGG​ACA​GTA​TC 3′	6,102	62	55
	R	5′ GAC​AAT​CTC​CTT​GCG​CTT​CT 3′	6,019	60	50

### 2.4 Data analysis

Microsoft Excel was used for data manipulation of raw Ct values collected from the qRT-PCR experiment. Statistical analysis of the data collected during the viability experiments was performed using either Microsoft Excel, RStudio Desktop (version ≥4.2.0), or GraphPad Prism 8.0.2 for Windows (GraphPad Software, Boston, Massachusetts, United States, www.graphpad.com). For the gene expression data analysis, we implemented the Livak formula (Livak and Schmittgen, 2001). Statistical testing on 2^−ΔCt^ values and the relative expression fold change (FC) were evaluated using the R application qDATA (https://github.com/A-Ionascu/qDATA). Graphical representations were created using GraphPad Prism. Statistical significance was considered at a *p*-value ≤0.05.

The genomic context of *CG18135* was visualized using JBrowse 1.16.10 accessed from Flybase (FlyBase Curators, 2004; Gramates et al., 2022). We used various tracks including BLAST, nucleotide view, gene span, RNA, transgenic insertions, and transcriptional regulatory regions (REDfly), as well as set highlight from view options. The cis-regulatory modules (CRMs) were estimated according to REDfly v9.6.2 (Rivera et al., 2019). Motif identification within the considered CRMs was performed using the XSTREME tool from the MEME Suite 5.5.4 (Grant and Bailey, 2021). For subsequent inquiries regarding the known functions of the proteins binding specific motifs, we referred to Flybase data (FlyBase Curators, 2004; Gramates et al., 2022).

## 3 Results

### 3.1 Viability experiment

In the first viability experiment, we collected a total of 117, 176, and 236 embryos corresponding to CantonS, *CG18135-P{lacW}*/TM6B, and *CG18135-P{lacW}*/GFP, respectively. After randomly placing individual embryos in the 24 available wells of each plate, we assessed their development until they reached the second or third instar larval stages. We recorded embryonic and larval lethality as well as larval viability (the number of observed living larvae) within each *D. melanogaster* line. We also assessed the fluorescent phenotype (green GFP or pale-blue auto-fluorescence) of viable larvae originating from the *CG18135-P{lacW}*/GFP line (Table 2). For embryos, we estimated the survival rate by subtracting the number of observed dead embryos from their initial total. Regarding larval lethality, it was mainly assessed based on the indirect sources of evidence found in each corresponding well, such as empty vitelline coats, moth piece residuals, foraging evidence, or, in some wells, their absence.

**TABLE 2:** This table provides the data collected in the first viability experiment. The number of deceased embryos (embryonic lethality), observed living larvae (larval viability), and presumably deceased larvae (larval lethality) are indicated. Within brackets, there are specified percentages of individuals ascribed to each assessed developmental stage relative to the initial number of embryos from each of the three *D. melanogaster* lines, control CantonS, *CG18135-P{lacW}*/TM6B, and *CG18135-P{lacW}*/GFP.

Development assessment	*D. melanogaster* lines		
	CantonS	*CG18135-P{lacW}*/TM6B	*CG18135-P{lacW}*/GFP
Total embryos	117 (100%)	176 (100%)	236 (100%)
Embryonic lethality	7 (6%)	69 (39%)	87 (37%)
Total larvae	110 (94%)	107 (61%)	149 (63%)
Larval viability	106 (90.6%)	72 (41%)	111 (47%)
Larval lethality	4 (3.4%)	35 (20%)	38 (16%)

For the CantonS embryos, we observed a mortality rate of ≈6%; thus, this value was used for correcting non-specific lethality and the estimation of the expected lethality in mutant lines. The observed mutant embryonic lethality was ≈37% (87 embryos) for the *CG18135-P{lacW}*/GFP line and ≈39% (69 embryos) for the *CG18135-P{lacW}*/TM6B line. The mortality rate of ≈6% in CantonS was considered when theoretically expected embryonic lethality was estimated in the mutant lines. For example, in the case of the *CG18135-P{lacW}*/GFP line, there are three theoretically expected descendant genotypes: *CG18135* ^*P{lacW}CG18135*^/GFP (50%), *CG18135* ^*P{lacW}CG18135*^ */CG18135* ^*P{lacW}CG18135*^ (25%), and GFP/GFP (25%). Under the hypothesis that the *CG18135* ^*P{lacW}CG18135*^ allele has no effect on embryonic lethality, only the GFP/GFP category, known to exhibit complete lethality during early embryonic stages, should contribute to the total embryonic lethality of roughly 25%, to which we have to add 4.5% (the correction relative to the CantonS mortality rate). The same logic is appropriate for TM6B/TM6B embryos.

**FIGURE 1:**
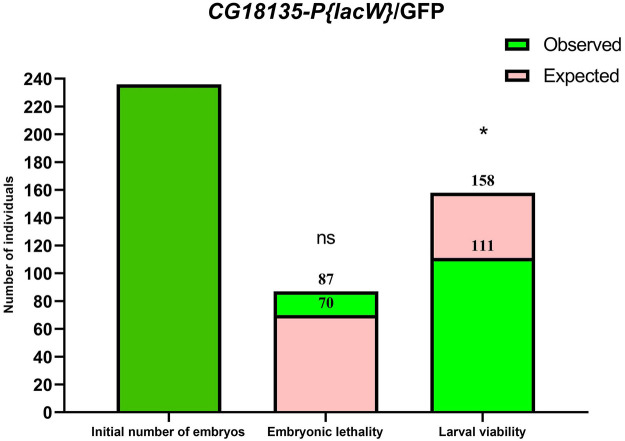
Bar plots of embryonic lethality and larval viability relative to the initial number of embryos considered for the *CG18135-P{lacW}*/GFP line. The observed total lethality of embryos (87 embryos) is compared to the expected 29.5% lethality (70 embryos). Likewise, the observed larval viability (111) is opposed to the expected viability (158). Both chi-square and Fisher’s exact tests failed to find statistical significance (ns, because calculated *p*-values are >0.05) for embryonic lethality, but they identified statistically significant differences for larval viability (* for 0.01 < *p* < 0.05).

In practice, statistical analysis (Figure 1) revealed no significant difference between the observed (87 embryos) and expected (70 embryos) embryonic lethality estimated for the descendants of the mutant *CG18135-P{lacW}*/GFP line (χ^2^ = 1.32, df = 1 and *p* = 0.2397 for the chi-squared test and *p* = 0.2688 for the Fisher’s exact test). It is plausible that among the dead embryos, there were some pertaining to *CG18135* ^*P{lacW}CG18135*^/GFP and/or *CG18135* ^*P{lacW}CG18135*^ */CG18135* ^*P{lacW}CG18135*^ genotypes, but we expect that the GFP/GFP genotype holds up the majority of embryonic lethality.

Regarding the observed embryonic lethality evaluated for the *CG18135-P{lacW}*/TM6B line, we also did not find statistical significance (χ^2^ = 1.780, df = 1 and *p* = 0.1822 for the chi-squared test and *p* = 0.2059 for the Fisher’s exact test).

In addition to the evaluation of embryonic lethality, we assumed that all the viable (remaining) embryos continued their development throughout the larval stages. Relative to the initial number of embryos, we found second and third instar larval viability of ≈41% (72 larvae), 47% (111 larvae), and 90.6% (106 larvae) for *CG18135-P{lacW}*/TM6B, *CG18135-P{lacW}*/GFP, and CantonS, respectively. These percentages were mirrored by larval lethality data of ≈20% (35 larvae), 16% (38 larvae), and 3.4% (4 larvae) collected for the same aforementioned lines. Similar to embryonic evaluation, if we take into account the CantonS larval lethality and accept that the *CG18135* ^*P{lacW}CG18135*^ allele has no negative impact on larval development and survival, then the expected larval viability for both mutant lines is approximately 70.5% (which is also the percent of expected viable embryos), from which we have to subtract 3.4% (observed larval lethality in the control line), thus obtaining a value of 67.1%. In this context, there are statistically significant differences between expected and observed larval viability for both *CG18135-P{lacW}*/GFP (χ^2^ = 5.252, df = 1 and *p* = 0.0219 for the chi-squared test and *p* = 0.0263 for the Fisher’s exact test; Figure 1) and *CG18135-P{lacW}*/TM6B (χ^2^ = 7.285, df = 1 and *p* = 0.007 for the chi-squared test and *p* = 0.0087 for the Fisher’s exact test) mutant lines. The lowered viability for *CG18135-P{lacW}*/GFP is not significantly different from that of *CG18135-P{lacW}*/TM6B (χ^2^ = 0.5272, df = 1 and *p* = 0.4678 for the chi-squared test and *p* = 0.4988 for the Fisher’s exact test). Moreover, the majority of GFP larvae, having the *CG18135* ^*P{lacW}CG18135*^/GFP genotype, completed their larval and pupal development and reached adult stages, similar to CantonS offspring.

**FIGURE 2:**
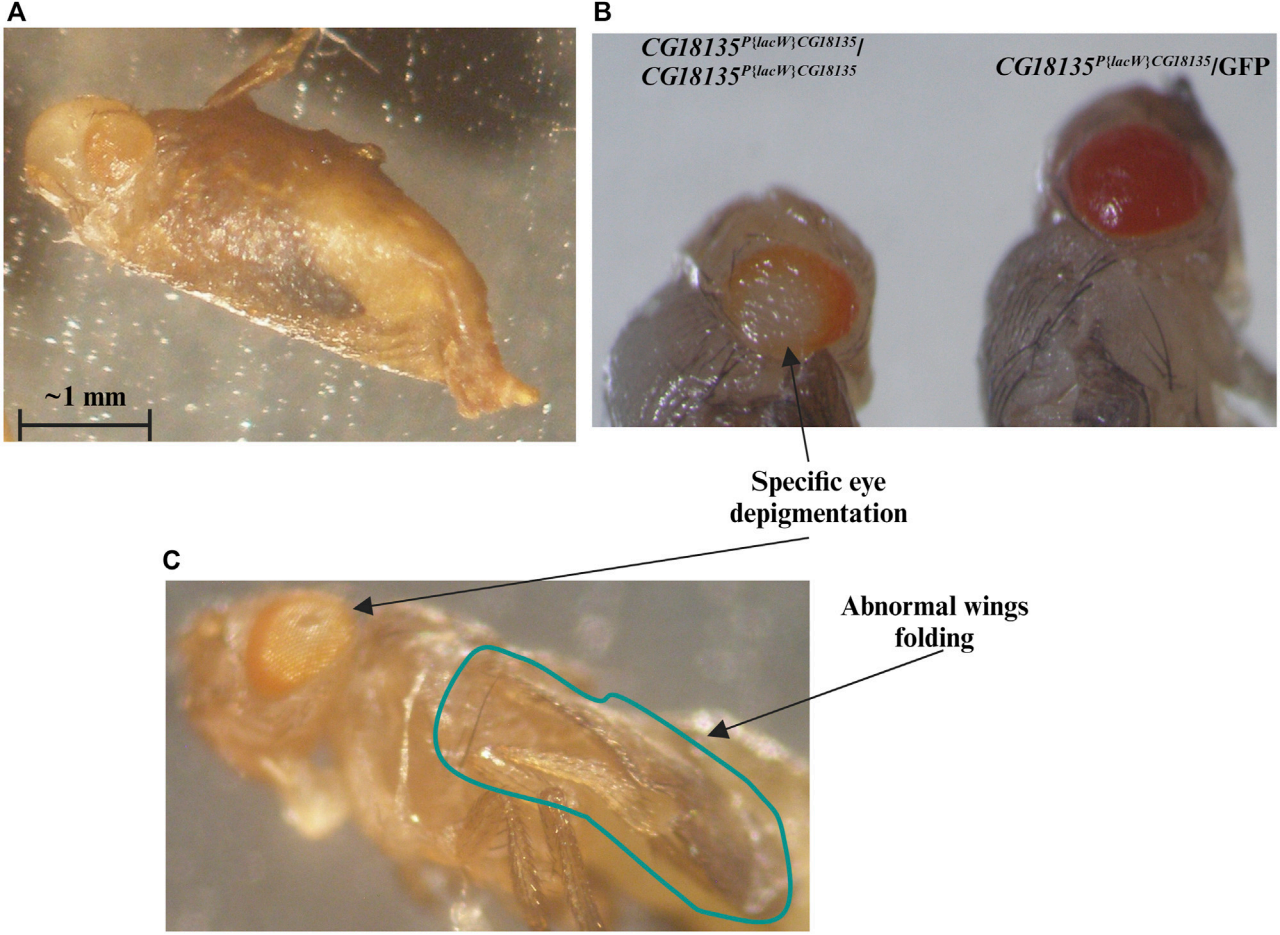
We highlight the evident mutant phenotype characteristic for the *CG18135* ^*P{lacW}CG18135*^/*CG18135* ^*P{lacW}CG18135*^ genotype. Most individuals that reach the end of the pupal development remain captive within the puparium or only succeed to partially emerge from it **(A)**. **(B)** We show *CG18135* ^*P{lacW}CG18135*^/*CG18135* ^*P{lacW}CG18135*^ and *CG18135* ^*P{lacW}CG18135*^/GFP individuals that reached the end of pupal development but were manually extracted out of the puparium. The eye depigmentation of the homozygous mutant is visible when compared with the pigmentation of the heterozygous mutant. The imago individuals also show a particular eye depigmentation pattern as well as persistent wing folding **(C)**. The horizontal bar shown in the figure indicates a length of 1 mm.

For the second viability evaluation, we focused on the *CG18135-P{lacW}*/GFP line and started with a total of 765 embryos. Batches of five randomly selected embryos were placed in each of the 24 wells of a given culture plate. When larval viability was estimated, we assumed by default the embryonic lethality of 37%, as previously estimated. Accordingly, we counted a viability of ≈43.8%, which is very close to our prior calculations. Out of the viable second and third instar larvae, we selected 104 non-GFP larvae (presumably with the *CG18135* ^*P{lacW}CG18135*^/*CG18135* ^*P{lacW}CG18135*^ genotype) that were individually placed in 48-well plates. Each well was monitored on a daily basis, and the developmental progressions were carefully noted. We found that ≈14.4% of larvae do not reach the pupal stage, while all the remaining larvae proceed at least until the late pupal stages. Compared with the initial number of larvae, around 65% of them get fully developed eyes and folded wings visible through the puparium. Out of the starting larval stage, at least 12.5% (13 individuals) attain the imago stage, and the corresponding adults either only open the puparium (Figure 2A, Supplementary Material S2) and are eventually partially getting out of it (nine individuals) or completely breach the puparium and land on medium (four individuals). Either way, the maximum lifespan of the partially or fully emerged imago is about 48 h.

### 3.2 Imago mutant phenotype

The escaper imago individuals of both sexes that harbor the *CG18135* ^*P{lacW}CG18135*^/*CG18135* ^*P{lacW}CG18135*^ genotype exhibit a series of noteworthy specific phenotypes in addition to early onset lethality. The first noted is a peculiar central depigmentation visible at eye level in both puparium captive and emerged adults, especially when comparing homozygous and heterozygous mutants (Figure 2). The homozygous mutant individuals have *w* ^*-*^ genetic background, and therefore, their eye color is caused by the *mini-white* ^*+*^ allele present in the *P{lacW}* transposon. Regarding the *CG18135* ^*P{lacW}CG18135*^/GFP genotype, since the GFP chromosome harbors two additional artificial P insertions, their eye pigmentation is considerably more intense than that of individuals with *CG18135* ^*P{lacW}CG18135*^/*CG18135* ^*P{lacW}CG18135*^ or *CG18135* ^*P{lacW}CG18135*^/TM6B genotypes. When naturally or manually puparium-freed imago individuals were available and subsequently monitored, we also noticed an abnormally persistent wing folding during the maximum lifespan of 2 days (Figure 2C).

In addition to these morphological phenotypes, we noticed that most adults fail to completely emerge from the puparium. Those who are successful are not able to perform locomotion and commonly adopt a stationary position with their legs oriented upward. Although not able to freely move around, such individuals manifest spasmodic leg movements that manifest in seizure episodes (Supplementary Material S3). As previously stated, we did not notice any viable adult older than 2 days.

### 3.3 *CG18135* gene expression in viable adults

**FIGURE 3:**
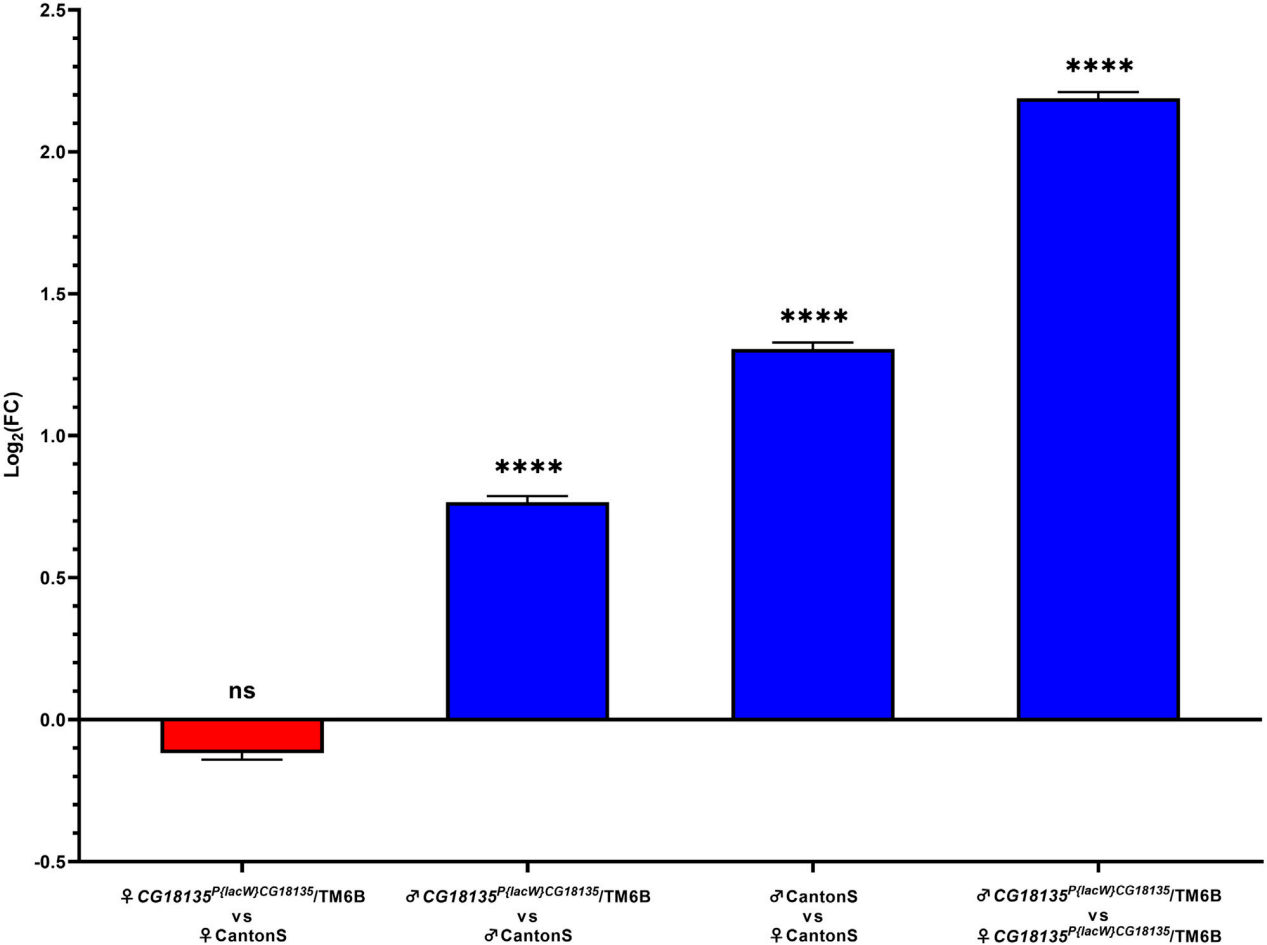
Relative gene expression analysis of *CG18135* in mutant *CG18135* ^*P{lacW}CG18135*^/TM6B and control CantonS male flies and female flies. The bars are in accordance with the log_2_FC values, and the mean ±SEM error bars are shown. Statistical significance was assessed using both Welch’s two sample *t*-test and the exact Wilcoxon rank sum test on 2^−ΔCt^ values, corresponding to each analyzed category. A statistically significant difference was considered at *p* ≤ 0.05 (ns > 0.05, **** <0.001).

Relative gene expression analysis was based on comparing FC values that were generated using the Livak method. Our data (Figure 3) show no significance for the gene downregulation in *CG18135* ^*P{lacW}CG18135*^/TM6B mutant female flies compared to CantonS control female flies (FC = 1.017) and a significant overexpression of *CG18135* in *CG18135* ^*P{lacW}CG18135*^/TM6B mutant male flies *versus* CantonS control male flies (FC = 1.850). Moreover, *CG18135* was significantly overexpressed in control male flies compared to control female flies (FC = 2.724), with a higher level in mutant male flies compared to mutant female flies (FC = 4.927).

Statistical testing was performed between each experimental group using either Welch’s two-sample *t*-test or the exact Wilcoxon rank sum test with the qDATA application (Ionascu et al., 2023). We used these two tests because of the distribution differences in 2^−ΔCt^ values corresponding to the experimental groups. Specifically, while the 2^−ΔCt^ distribution for the heterozygous mutant female flies was strongly positively skewed, the data calculated for the other groups showed a parametric distribution. Moreover, the variance of 2^−ΔCt^ values calculated in the four experimental groups was different within one grade of magnitude, thus suggesting not equal variances. The results are presented in detail in Table 3.

**TABLE 3:** Results of statistical testing on 2^−ΔCt^ values calculated for the mutant *CG18135* ^*P{lacW}CG18135*^/TM6B male and female genotypes and control CantonS groups. Data were obtained using the qDATA application.

Comparative analysis	Welch’s two sample *t*-test		Exact Wilcoxon rank sum test	
	t statistic	*p*-value	W statistic	*p*-value
*CG18135* ^*P{lacW}CG18135*^/TM6B ♀ vs CantonS ♀	−0.826	0.415	294	0.228
*CG18135* ^*P{lacW}CG18135*^/TM6B ♂ vs CantonS ♂	6.648	2.34 × 10^−8^	654	5.396 × 10^−8^
CantonS ♂ vs CantonS ♀	9.138	1.4 × 10^−10^	715	5.218 × 10^−13^
*CG18135* ^*P{lacW}CG18135*^/TM6B ♂ vs *CG18135* ^*P{lacW}CG18135*^/TM6B ♀	15.584	3.202 × 10^−16^	729	1.027 × 10^−15^

### 3.4 Genomic context of *CG18135*

**FIGURE 4:**
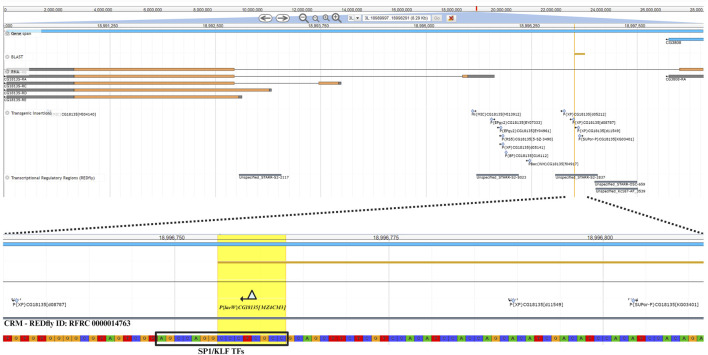
The upper section offers a panoramic view of the *CG18135* gene and overlapping genomic features provided by specific JBrowse annotation tracks, such as gene span, RNA, transgenic insertions, and transcriptional regulatory regions. In the lower section, the *P{lacW}* ^*CG18135.MZ4CM3*^ insertion, the duplicated octet, and its type II orientation are highlighted. The SP1/KLF TF binding site found inside the CRM with the REDfly ID:0000014763 (this CRM also appears under the name Unspecified_STARR-S2-2837) is indicated, as it is located in the innermost vicinity of *P{lacW}*.

The nucleotide sequence next to the *P{lacW}* ^*CG18135.MZ4CM3*^ insertion was aligned against the *D. melanogaster* genome (r6.54) using BLAST from FlyBase (FlyBase Curators, 2004). The result was visualized in JBrowse using specific track selection and a highlight on the CCCTCGCC sequence (this specific nucleotide octet is duplicated upon *P{lacW}* insertion), representing the transposon target site with the genomic coordinates 3L:18996755–18996762 (Figure 4). The insertion has a type II orientation, meaning that its sense strand is collinear with the minus genomic strand, and it is the only *P{lacW}* hit reported for *CG18135*. Otherwise, only *P-* , *PBac-* and *MiMIC*-derived transposons are reported in FlyBase (FlyBase Curators, 2004; Gramates et al., 2022), such as *P{EPgy2}*, *P{RS5}*, *P{XP}*, *P{EP}*, and *P{SUPor-P}*. Twelve transposon insertions, including *P{lacW}* ^*CG18135.MZ4CM3*^, span the genomic coordinates 3L:18995536–18996803, which define a genomic region encompassing fragments from the first intron of CG18135-RB and the 5′UTR of CG18135-RA. According to the REDfly annotation track, there are four distinct CRMs that overlap or border this genomic region. Out of them, the one denoted REDfly:RFRC:0000014763 (CRM14763) overlaps the insertion sites of four previously reported *P*-derived transposons and *P{lacW}* ^*CG18135.MZ4CM3*^. We recovered the CRM14763s-specific sequence and input it to XSTREME, a specific tool from the MEME Suite that is able to perform broad motif analysis on sequences with scarce motif sites using generic eukaryote or *D. melanogaster* restricted motif databases. The XSTREME analysis emphasized that CRM14763 harbors specific binding sites for SP1/KLF transcription factors (TFs), MADF–BESS domain transcription regulators, and nuclear receptor (ligand-dependent) TFs.

The SP1/KLF TFs’ binding motif has the AGCCAGGCCCTCGCC nucleotide sequence and encompasses the transposon target sequence (the CCCTCGCC subsequence).

## 4 Discussion

In this study, we investigated the mutant phenotypes of offspring from *CG18135-P{lacW}*/GFP and *CG18135-P{lacW}*/TM6B lines. We found that, for *CG18135* ^*P{lacW}CG18135*^/*CG18135* ^*P{lacW}CG18135*^ mutants, the larval and pupal developmental stages were strongly associated with lethality. To the best of our knowledge, this is the first viability analysis of any *CG18135* allele in *D. melanogaster*.

The lack of statistically significant differences between the observed and expected lethality of mutant embryos suggests that the *CG18135* ^*P{lacW}CG18135*^ allele is not associated with embryonic lethality. This result could be attributed to the parental delivery of functional CG18135 proteins either in the unfertilized egg (Fisher et al., 2012) or via the seminal fluid (Findlay et al., 2008), fulfilling the CG18135 protein requirements in early embryonic stages.

Previous proteomics studies identified increased CG18135 protein levels in *D. melanogaster* larvae, pupae, and adults compared to embryos (Brown et al., 2014; Casas-Vila et al., 2017). In addition, high expression of *CG18135* was found in the Bolwig’s organ in larvae (Fisher et al., 2012). The corresponding protein was identified within the plasma membrane of *D. melanogaster* adults’ heads (Aradska et al., 2015). Taken together, these data suggest that the CG18135 protein requirement could be higher in larvae, and in the absence of wild-type protein production, the maternal or paternal CG18135 delivery in embryos is not sufficient to support normal development, thus increasing larval lethality.

We observed that a small percentage of *CG18135* ^*P{lacW}CG18135*^/*CG18135* ^*P{lacW}CG18135*^ pupae developed to the adult stage. The CG18135 protein participates in myosin filament binding within muscular tissues and cell division (Liu et al., 2008). Therefore, it is expected that the protein requirement increases during pupal development, which implies organogenesis and high rates of cellular division. Our previous preliminary data revealed that the pupal lethality of CantonS and individuals heterozygous for the *CG18135* ^*P{lacW}CG18135*^ allele is ≈6.7% and ≈10.3%, respectively. For *CG18135* ^*P{lacW}CG18135*^/*CG18135* ^*P{lacW}CG18135*^ mutants, almost all the surviving individuals that get through the embryonic and larval stages would die inside or attached to the puparium.

These results reveal two major lethality thresholds in the development stages: one in the early larval stage (but we do not exclude a certain degree of mortality in the late pre-larval embryonic stage) and the second in the late stages of pupal development. The latter could be considered partial lethality since rare imago escapers are active for a maximum of approximately 2 days, but beyond that age, the lethality induced by the *CG18135* ^*P{lacW}CG18135*^/*CG18135* ^*P{lacW}CG18135*^ genotype is absolute.

**FIGURE 5:**
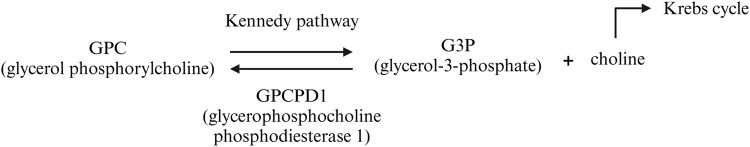
Biochemical pathway of GPC catabolism mediated by the GPCPD1 protein (in humans). It is possible that a similar mechanism is also true for the CG18135 protein in *D. melanogaster*.

We hypothesize that the *CG18135* gene participates in various biological and molecular processes in *D. melanogaster*, similar to its structural orthologous gene in higher eukaryotes, *GPCPD1*. In humans, *GPCPD1* knockdown of malignant cells showed an increased intracellular glycerophosphocholine/phosphocholine (GPC/PC) ratio, a decreased lipid metabolism, and cell migration inhibition (Steward et al., 2012). In higher eukaryotes, GPCPD1 facilitates GPC degradation, an essential phospholipidic membrane constituent (Eibl, 1980; Okazaki et al., 2010), into glycerol-3-phosphate (G3P) and choline (Steward et al., 2012). In eukaryotes, the G3P requirement is obtained from glycolysis (Li et al., 2019; Nguyen et al., 2019), and together with choline, it is further used for GPC synthesis via the Kennedy pathway (Veldhuizen et al., 1989; Fagone and Jackowski, 2013). These observations suggest that the CG18135 protein may be involved in the regulation of a negative feedback loop between G3P, choline, and GPC (Figure 5). Furthermore, choline contributes to the *de novo* synthesis of phosphatidylcholine and phosphatidylethanolamine membrane constituents via the Kennedy pathway (Gibellini and Smith, 2010; Steward et al., 2012) and participates in the Krebs cycle (Steward et al., 2012), being an essential component in energy metabolism.

The observed phenotypes of homozygous mutant imago escapers are consistent with the proposed involvement of the *CG18135* gene in either muscular activity or energy metabolism.

The participation of *CG18135* in embryonic myosin filament binding has been previously demonstrated (Liu et al., 2008). Being localized in the cellular membrane vicinity, *Myo10A* participates in myosin filament binding during embryogenesis together with several cargo proteins, including DE-cadherin, alpha-tubulin, Katanin-60, Milton, atypical protein kinase C (aPKC), NEDD4, and the CG18135 protein. This interaction might be, at least partially, responsible for the unsynchronized movements observed in mutant adult escapers, as an abnormal delivery of the CG18135 protein could result in muscle contraction defects. In addition, the structural orthologous gene *GPCPD1* is associated with muscle development in mice (Okazaki et al., 2010; Cikes et al., 2021). Okazaki et al. (2010) showed that *GPCPD1* gene expression was downregulated in atrophied skeletal muscles, and *GPCPD1* gene knockout enhances myoblastic differentiation and limits the progression of skeletal muscle atrophy (Okazaki et al., 2010). Thus, a lack of sufficient CG18135 protein could increase the lethality rates in larvae or pupae, as observed in our study.

A recent study suggests that the inactivation of *GPCPD1* also results in early muscle aging due to GPC accumulation (Cikes et al., 2021). More specifically, the “aged-like” muscles of *GPCPC1* deficient mice were caused by abnormal glucose metabolism and not by muscle development *per se* (Cikes et al., 2021). Interestingly, increased GPC levels were found only in the muscle (but not in the global lipidome) and did not occur in *GPCPD1* deficiency in other tissues, such as fat, brown fat, or liver (Cikes et al., 2021). These results suggest that *GPCPD1* is associated with glucose metabolism within the muscle tissue.

In addition, from the spasmodic leg movement of imago escapers, we observed a general lack of activity, which could be caused by either an abnormal energy metabolism or a decreased response to environmental stimuli. We would rather overlook the latter, as *CG18135-P{lacW}*/GFP is derived from a line that did not show any phenotypes that might suggest an abnormal response to the stimuli. Considering the previously discussed functions of *GPCPD1* in muscle contractions and energy metabolism, the sedentary phenotype might be caused by a lack of global energy availability in the imago escapers or by a lack of coordinated muscle contractions that require ATP. These phenotypes were evident in imago individuals, but we did not notice such phenotypes in larvae. Although more investigations are required, preliminary data might suggest that CG18135 protein necessity is higher in the later stages of development. Further experiments might not only focus on the expression of the *CG18135* gene in mutant larvae or pupae but also on the GPC/PC ratio in mutants compared to wild-type.

Considering the high lethality of *CG18135* ^*P{lacW}CG18135*^ */CG18135* ^*P{lacW}CG18135*^ mutant flies prior to the adult stage, we decided to evaluate the *CG18135* gene expression in adult flies, both in male *versus* female groups and in mutant *versus* control groups. Following the quantification of *CG18135* gene expression in different *D. melanogaster* adult groups, we found that *CG18135* ^*P{lacW}CG18135*^/TM6B mutant female flies and CantonS control female flies displayed a similar expression level, even if the mutant female flies had only one functional allele of *CG18135*.

Interestingly, we found that *CG18135* was significantly overexpressed in heterozygous mutant male flies compared to control male flies. It is plausible that in the absence of one functional allele, there might be an increase in the GPC/PC ratio (Steward et al., 2012). In this context, in order to reduce the GPC/PC ratio, an upregulation of *CG18135* and correspondingly greater protein production are necessary. The biochemical pathways involving phospholipids are complex, and because we did not observe similar expression profiles in mutant female flies compared to control female flies, we consider that it is not possible to infer a definitive conclusion.

In male flies, the CG18135 protein is present in the seminal fluid of *D. melanogaster* individuals at mating (Findlay et al., 2008), which could partially explain the overexpression of the *CG18135* gene in mutant or wild-type male flies compared to female flies. Additionally, the reproductive system of male flies is associated with intense cellular division and cellular differentiation during spermatogenesis (Fabian and Brill, 2012). These processes are dependent on the availability of glycerophospholipids and may influence the expression of *CG18135*.

The observed *CG18135* overexpression in male flies compared to female adult flies is consistent with previous transcriptomics studies (Brown et al., 2014; Casas-Vila et al., 2017). The *CG18135* overexpression in heterozygous mutant male flies *versus* heterozygous mutant female flies (FC = 4.927) could be caused by a combination of factors that drive overexpression in mutant male flies compared to control male flies (FC = 1.85) and in control male flies compared to control female flies (FC = 2.724).

Genomic context analysis showed that the P{lacW} insertion in *CG18135* resides adjacently to a putative binding site for SP1/KLF TFs. Several members of this family of zinc finger proteins, such as Sp1, buttonhead, and cabut (cbt), act as activators or repressors of various developmental processes involving head structures and sensory organs (Brown et al., 2005). If the respective binding site is not efficiently accessed by cbt, a series of downstream interactions could be influenced, including dorsal closure and neuronal development in embryos, wing disc morphogenesis, muscle degeneration, and energy metabolism (Bejarano et al., 2008; Belacortu et al., 2011; Rodriguez, 2011). Abnormal cbt activation might partially explain the specific wing phenotype in mutant imago escapers, but it could also influence the mechanism of flight and jump muscle control as well as the regulation of the number of tergal depressors in the trochanter (TDT) jump muscles (Giedd, 2018). Considering that cbt takes part in energy metabolism via a regulatory link between sugar sensing and the circadian clock (Bartok et al., 2015), cbt interaction with the *CG18135*-located binding site might further aggravate the sedentary behavior or sporadic movements of mutant adult escapers.

In humans, the homologs of *cbt* are *Krüppel-like factor 10* (*KLF10*) and *KLF11* (Bartok et al., 2015). Both are part of a TF family that regulates CG-rich promoters (Lomberk et al., 2013) and influences energy metabolism. *KLF11* is upregulated during starvation (Zhang et al., 2013), while *KLF10* is overexpressed in response to high sugar levels (Iizuka et al., 2011) and is considered a true *cbt* functional ortholog (Bartok et al., 2015). These TFs are also involved in TGF-β-mediated cell proliferation, differentiation, and apoptosis (Lomberk and Urrutia, 2005).

In our view, the mere presence of *P{lacW}* ^*CG18135.MZ4CM3*^ might exert profound effects on the overall transcription regulation of *CG18135* since CRM14763 is split and various neighboring CRMs that could share functional roles are forcibly separated.

In this study, we characterized mutant phenotypes produced by the *CG18135* ^*P{lacW}CG18135*^ insertional allele for the first time in *D. melanogaster*. We performed several viability assessments, which allowed us to establish that the early larval and late pupal developmental stages are critical for lethality onset. Rare imago escapers that live a very short life exhibit very specific phenotypes, affecting the eye and wing morphology as well as their locomotor proficiency. These severe phenotypes are probably the consequence of the regulatory perturbations that are likely to be caused by the insertion of the *P{lacW}* ^*CG18135.MZ4CM3*^ transposon inside a group of CRMs containing binding sites recognized by wing and energy metabolism-specific, as well as ubiquitous, TFs. The heterozygous mutant flies are normally developing, but we found that the gene expression of *CG18135* is significantly different in male flies compared to female flies and in mutant flies compared to control male flies. Our findings support the essential role of the *CG18135* gene in *D. melanogaster* and define a novel experimental platform for modeling the interactions of the *GPCPD1* structural and functional orthologous genes in higher eukaryotes.

## Data Availability

The datasets presented in this study can be found in online repositories. The names of the repository/repositories and accession number(s) can be found at: https://www.ncbi.nlm.nih.gov/nuccore/HQ695001.1/.
